# Selections from the Foreign Periodicals

**Published:** 1846-04

**Authors:** 


### CIRCUMSPECTIVE REVIEW.

### Selections from the Foreign Periodicals.

### Treatment of Cutaneous Diseases.

1. *By Alkalis.*—M. Devergie states that alkalis are highly beneficial in skin-diseases, especially those of the *lichenoid* form. Their *internal* exhibition is indicated whenever the cutaneous affection is connected with gastralgia dependent upon acid secretion, whatever may be the anatomical character of the eruption: and their *external* employment is of service in papular and squamous diseases. Completely successful in the first of these categories, they are less so in the second; and are only to be used when the disease is chronic in either. The bicarbonate of soda or potass, may be given in moderate doses (ʒj. ad ℈j. per diem), either in water, Vichy water, or a slightly bitter and stimulant tisane, (*Bic. Pot.* 5 *parts. Infus. of Lime-leaves* 125 *pt. Syrup of Mallows* 45 *pt. Spt. of Mint gtt.* 25. Take two or three spoonfuls per diem.) The digestive functions become re-established, the appetite especially increasing in vigour. If the doses given are very large, the fibrine of the blood becomes diminished in consistency, disposing to the production of glandular swellings and passive hæmorrhages. *Alkaline ointments* are often employed too strong, and M. D. only orders ½ or one part of *Subcarb. soda* to 30 parts of lard in cases of *lichen*, and from 1 to 2 parts to 30 in *psoriasis, lepra, &c.* The following is an alkaline liniment he employs. *Carb. Sod. or Potass* 30 *pts. Ol. Oliv.* 125 *pts. One yelk of an egg.* M. When we have to do with an affection of the hairy scalp, we may use a stronger ointment by increasing the proportion of soda (4 or 6 pts. to 30), substituting subcarb. potass for it, or adding to either its own weight of slaked lime. When employed in *lichen*, for which these remedies are pre-eminently adapted, the itching is very speedily allayed; but they must be continued a certain time after the disappearance of the papulæ, or a relapse will occur.—*Bulletin Generale.*

2. *By Caustics.*—M. Duchesne-Duparc has recently published an interesting paper upon the application of caustics to the skin. Some of the forms of *erythema* among the inflammatory diseases, derive great benefit from the nitrate of silver. Thus a pencil, or solution (5 or 10 parts to 30 of water,) may be usefully employed in the *intertrigo* of the ears of children, when ulcerations have commenced, whose surfaces are covered with a thick coagulum. If care be taken not to destroy commencing cicatrization, it may be frequently re-applied with great success. In *chilblains* and *chronic erythema,* applying the *lapis infernalis* to the part, and some little distance beyond, is successful. In *erysipelas,* when we wish to prevent the invasion of important parts, or circumscribe its extension, we must encircle it with the nitrate of silver, extending beyond its margin, or if the erysipelas be but small in extent, we may cover the whole surface with the same. It requires frequent renewal and is more efficacious, as it is applied nearest the commencing point of the disease, and effectually influences that part. In *traumatic erysipelas,* the actual cautery, which at a white heat causes little pain, arrests the disease at once, and leaves neither suppuration or scar. When the patient will not submit to this, the frequent renewal of the nitrate of silver lotion must be substituted.

The various forms of *herpes* yield readily to caustics. When the vesicles are few and widely separated, each may be opened separately, the matter it contains, if abundant, squeezed out with a piece of lint, and the interior very lightly touched with a fine pencil of *lapis infernalis*. When the vesicles are confluent or agglomerated, it is better to cover them with a thin layer of nitrate of silver, or apply a concentrated solution of this substance to them. Cauterization carefully performed (1) abridges the duration of the disease: (2) prevents the formation of excoriations and eschars, which follow herpetic eruptions, (especially in old men), when left to themselves, and particularly when they are seated on the posterior part of the trunk; and (3) in the great majority of cases, prevents the pains, which are often severe, and prolonged for a considerable time after the disappearance of the eruption. A marked proof of the benefit of this treatment is derived from the fact that, slightly touching the red spots which precede the vesicles prevents the development of these, although it does not always relieve the severe pains which attend them. We agree with M. Rayer, that slight and discrete herpes may be left to itself: the nitrate being, however, always a precious resource when eschars are to be feared in one or more groups seated on the face or trunk.

Although in the exanthematous form of *urticaria* caustic is not indicated, it is the best of means for the relief of the swelling and itching in the form which appears in detached tumefactions, resembling the stings of insects or plants.

M. Velpeau states, that when applied within three days of its appearance, caustic will at once arrest the progress of a *furunculous tumour*, which, however, Rayer denies. M. D's experience is in favour of the former opinion; and he explains the want of success attending the use of caustic by the timid manner in which it is applied. A concentrated solution of the nitrate, or the moistened lapis infernalis, should be employed. It is not meant to say that all cases will yield to this, as some may require constitutional treatment.

It is certain that the development of the pustules of *small-pox* may be arrested by caustic during the two or three first days of their progress. M. Bretonneau's mode of applying it is perhaps the preferable one; viz. removing the point of the pustule with a gold or silver needle, and cauterizing the bottom with a pencil of lapis infernalis, or with a probe dipped in powder of the same substance. When *vaccination* has been performed in an improper situation, the abortion of the pustule may be brought about by the same means.

The obstinacy of *favus* is well known, and the efficacy of caustic is explained by the fact, that the disease is maintained by a vegetable parasite seated in the epidermis. Here *Ammonia* is the best caustic (*suet, and liquor. ammon. eq. p.; or ol. oliv* 1 *part, ammonia* 1 *or* 2 *pt.*). In young persons and adults the application must be retained for an hour; but in very young children, the proportion of ammonia and period of its application must be both diminished. In all cases the whole disease must be brought away, or a fresh crop will soon appear.

In *dartrous* affections caustic is a valuable means. Thus, in *pityriasis, psoriasis, lepra, icthyosis, &c.* the vitality of the surface undergoes a favourable modification by the use of mild caustics. In *eczema,* when the patches are circumscribed and of old date, the more powerful caustics are required, such as the acid nitrate of mercury or muriatic acid. In *impetigo* caustic is only proper for old, isolated patches. We must not use caustic at the commencement of any form of *acne,* but after a while, when the follicles become distended or burst, and a number of little diffused phlegmons, which equally resist antiphlogistics and astringents, result, the application of a saturated solution of *sulphuret of potass* to the point of the tubercle is highly useful. The application is to be retained for 15 or 20 seconds, and after a quarter of an hour, any pain or irritation it may have given rise to, is to be allayed by calming lotions. To be of use, this application must be continued for a long period, the evening being the best time of using it. Very obstinate cases of *mentagra* and *couperose* have yielded to it when they have defied all other means.

After alluding to the use of caustics in cancer, lupus, scrofula, and the syphilides, M. D. concludes with these general rules.

1. When the skin is thin and abundantly supplied with vessels and nerves, the mildest caustics must be used. This is especially the case with the face, and the nitrate of silver there usually answered all the requisite indications. 2. We must not attempt to cure the disease alone by caustics, except when it possesses no depuratory character, is connected with no diathesis, and can be looked upon as an isolated, accidental, morbid centre. 3. Caustic is no less useful when it does not alone suffice for the cure. Its use in reviving a languid inflammation, in stimulating flabby, inactive tissues, in repressing exuberant growths, and in remedying disorders of the innervation of the parts, is very great. 4. As each separate caustic acts upon healthy tissues differently as regards its intensity, mode of influence, and re-action, one must not be indiscriminately employed for another. 5. The mode of cauterizing varies according to the object we have in view. If it is a mere adjuvant the application should be superficial and limited. If it is an essential means of cure, its action should involve all the diseased parts, and often extend beyond them.

---

### On the Use of Kermes Mineral in Diseases of the Respiratory Organs. By *Dr. Herpin* of Geneva.

Dr. Herpin has diligently observed the effects of this medicine during eight years, and has arrived at some interesting conclusions respecting it. If we consult dictionaries, dispensatories, &c. we find this substance stated as being chiefly indicated in chronic and suffocative catarrh, humid asthma, and at the termination of pertussis and pneumonia, especially in aged persons. Dr. Herpin reports differently. He says he has never seen its use followed by even temporary benefit in the latter stages of *pneumonia* in the aged, when the mucous *râles* are abundant, and asphyxia imminent. Given too at the commencement of pneumonia following capillary bronchitis it is far inferior to tartar-emetic: and alterative doses of this same remedy are also far superior to it in the capillary *bronchitis* of old persons and children. The same want of success attended its trials in asthma and pertussis.

But when the disease, instead of being situated in the parenchyma and smaller bronchi, occupies the *larger passages* the result is very different. Laennec and all those who have come after him have repeated the erroneous statement, that *bronchitis*, from its very commencement, when a mere coryza, imparts to the ear a loud *râle*. Dr. Herpin some time since shewed that bronchitis at the upper part of the tube, accompanied by considerable secretion, gave no auscultatory sign whatever. It is in these cases, where no anormal sound exists, that *Kermes* is so useful, and is most so in the acute stage. He does not mean to say that it will arrest the progress of every pulmonary disease beginning without auscultatory signs, for hooping-cough, pneumonia and phthisis are among such. The catarrhs commencing at the upper part of the tube are soon arrested; but if *râles* announce the supervention of deep-seated bronchitis, other medicines must be resorted to. In *tracheitis*, (denoted by pain opposite the top of the sternum, sometimes difficult deglutition, a hoarse, tearing paroxysmal cough and hoarseness), the Kermes is still more indicated. No other medicine produces so rapid an effect in *laryngitis*, a few ¼-grain doses often removing the hoarseness in a few hours, when the disease is recent. In this way it is of great service to singers. *False-croup* is very advantageously treated by it, and if seen early enough, it may be of good service in the true disease. *Chronic laryngitis*, when not dependent upon phthisis, may be benefited by this medicine, but in an inverse proportion to its duration. Even when it does not cure it (for relapse is very frequent) it gives at least great relief. In the only case of *thymous asthma*, Dr. H. has had an opportunity of trying it, and which was a very bad one, it succeeded completely. In affections of the *pharynx* no success followed the use of the medicine, unless indeed these were connected with disease of the larynx. A frequent cause of *deafness* is a catarrhal condition of the extremity of the Eustachian tube, and in this case the Kermes effects a cure if the deafness has not existed beyond some weeks, and even alleviates it frequently when of very old standing. From his observations, Dr. H. believes he may deduce the conclusion, that *Kermes is to some extent a specific for the affections of the upper portions of the air-passages.*

The *dose* has varied from 1 to 12 grs. in the 24 hours. M. H. has never exceeded the latter quantity, and, as a general rule, from 3 to 6 grains suffice. If we except infants less than 1 or 2 years old, the dose need not be much varied on account of age, children tolerating the remedy almost as well as adults. It may be given in an emulsion, powder with sugar, lozenges or pills. At the commencement of the affection, or when the respiration is much oppressed, it is desirable to excite vomiting. Three grains will certainly effect this as will sometimes one or two in adults, and half a grain in children. To avoid purging or vomiting, we should give only very small doses and after meals. When tolerance is once established, the Kermes does not irritate the stomach again. When first given it causes a sense of heat and dryness in the throat, which soon becomes relieved by an increased humidity and expectoration. Dr. Lombard, who has employed this medicine with excellent effects, has often observed rose-coloured streaks in the expectoration; but these soon disappear.

### Carbonate of Iron in Menorrhagia, &c.

Dr. Malherbe observes, that believing in the emmenagogue character usually attributed to preparations of iron, he had never ventured to use them in uterine discharges, until he had perceived a case of menorrhagia occurring in a chlorotic girl, and advantageously treated by chocolate-iron, recorded in Trousseau and Piodon's *Treatise on Therapeutics*. Since that time, he has repeatedly used the subcarbonate of iron with the best effects. He selects for description thirteen cases of menorrhagia, occurring in women of very different ages and temperaments, and in whom the only symptom in common was an abundant loss of blood at regular or irregular menstrual periods. Acute, as well as chronic cases yielded to the subcarbonate, which was given in doses of from ½ to 1 ℈ at varying periods of frequency. He concludes, "That the subcarbonate of iron is not an emmenagogue, but, on the contrary, arrests or moderates the menstrual flux, arrests uterine hæmorrhages, and is in fact one of the most powerful hæmostatic means we have at our disposal."—*Journal des Connoissances.*

### Quinine in Epilepsy.

It is to be remembered that epileptic paroxysms, without observing a regularly intermittent course, nevertheless retain at epochs more or less distant from each other. Quinine, therefore which generally succeeds so well in periodical neuralgiæ, even when these are not strictly intermittent, deserved at least a trial among the multitudinous remedies which have been proposed. M. *Piorry*, at *La Pitié*, has for a long time employed it, and has cured a certain number of patients hitherto reputed incurable, these patients having been retained long enough in the hospital to prove that the course was not merely temporary.

The mode of administering the remedy is of consequence, for the dose must be large. Begin with 18 grains *per diem,* and gradually increase this until from 50 to 70 grains *per diem* are given. The medicine has to be continued for a very long period, as the cure is always slow, and cannot be always pronounced definitive until months or even years have expired.—*Gazette Medico-Chirurgicale.*

(In a disease so distressing and so intractible as epilepsy, any means of cure, even although not universally applicable, will be gladly resorted to. But we must confess that doses of Quinine so large as the above, and continued for months, seem to us unadvisable. The reader will find in the 10th Vol. of the Memoires de l'Academie (*Med. Chir. Rev. No.* 78, *p.* 306) a paper from the pen of M. *Melier,* exposing the dangerous and even fatal effects of the large doses of Quinine sometimes employed in France.—*Rev.*)

---

### M. Roux on Excision of the Elbow Joint, &c.

M. Roux, one of the earliest performers of this operation, made a few remarks upon it in a recent clinical lecture.

The case of the young woman upon whom I am about to operate presents some peculiar features, which have influenced me in preferring excision to amputation. She is the subject of white-swelling in each elbow, and, at the time of her admission, the left one had already become anchylosed at an obtuse angle, so as to be useless to her. The disease on the right side, after making much progress, has become stationary, and, although much pus is yet discharged from fistulous openings, it is possible that anchylosis of this arm might also be produced. But a more helpless and unfortunate condition than that resulting from such a double anchylosis can hardly be imagined, and, to prevent this, I have recommended excision of the enlarged extremities of the bones. Those who are not familiar with the operation, and are unacquainted with the results which have attended its performance, may inquire in what manner it can conduce to this patient's benefit; and it is, therefore, of importance that I should state, I have never known an anchylosis follow excision. The bones of the arm, by reason of a species of false joint being formed, invariably retain a more or less considerable degree of mobility upon the humerus, so that the patient is enabled to execute many of the normal movements of the limb. In proof of the considerable degree of usefulness of the extremity that is attainable, I may cite the case of a patient upon whom I operated seven or eight months since, who is able to take up the various objects of ordinary use, carry his hand to the mouth, and, in fact, perform various and extensive movements with it. I have seen many curious examples of persons who have been enabled to undertake occupations requiring both dexterity and strength.

In the great majority of cases it is in order to arrest the progress of the disease called *white-swelling* (vague as is this term, it is preferable to any of those which have been proposed in lieu of it,) that excision is practised; but there are other circumstances, as gun-shot wounds, &c., in which it may be required. There are cases of this disease in which the soft parts surrounding the joint are affected rather than the ends of the bones themselves; but, even in such cases, in which the bone is not the seat of the principal changes, the operation is yet very useful—the fungoid condition of the soft parts disappearing after its performance.

Although never performed so frequently as at the present period, this operation is not a new one; but we have no particular account of the mode of its performance in former times. Even now it is by no means generally adopted, and there are surgeons, both in France and other countries, who do not admit that excision is ever advantageous. Indeed, in France, the country of its revival, very few surgeons are in favour of it, and it is performed much more frequently in Germany. Perhaps M. Textor, of Wurtzbourg, has had recourse to excision more frequently than any other practitioner, and that not only in disease of the small and medium-sized joints, but also in affections of large ones, such as the knee. I have performed excision a great number of times, but my operations have been far less frequent than those of M. Textor. Moreover, warm partisan as I am of excision, I should not like to perform it upon the knee, and should not be without some fears of the possible results of such an operation. When M. Textor was in Paris, three or four years since, we compared the results we had obtained ; and, it is a curious fact that, we each reckoned up fourteen cases of excision of the elbow; and of these fourteen, we both had lost four patients.

The result of my experience has shown me that, although excision of the elbow is a long and laborious operation, the large and deep wound which it leaves unites entirely, or nearly so, by the first intention: and, even when an extensive suppuration is set up, it is rare that any ill consequences follow. Statistics prove to us that there are much fewer deaths after resections than after amputations. (?) Observe the enormous difference between the two operations. Amputation deprives the patient of his limb, and exposes him always to more or less danger. Resection is a somewhat longer, more painful, and more difficult operation, but it preserves the limb, permits eventually more or less extensive movements, and presents very few unfavourable chances. As we have already said, the elbow is of all joints that upon which resection can be performed with most advantage, and least danger. The tibio-tarsal is the most favourable joint of the lower extremity for the operation.

But although resections of the lower extremity are much more dangerous than those of the upper, they should not be proscribed in all cases. I have seen resection of the upper portion of the femur performed with success, as also of the bones constituting the knee-joint. Another reason, besides the danger, why I am less disposed to approve of these resections, of the bones of the lower extremity, is that one of their especial functions is to support the weight of the body. These joints then require, on the one hand, great mobility, and, on the other, great solidity and power of resistance. Thus, for example, a patient upon whom a resection of the head of the tibia has been performed, and in whom a complete anchylosis has not supervened, is in a worse condition than if he had undergone amputation of the thigh. A lower extremity, without complete anchylosis, would furnish a very insecure support. I have performed the operation upon the knee but once, and that upon a patient who strongly insisted upon it. I found it impossible to maintain the leg extended on the thigh; and this leads me to observe that, in the persons I saw in Germany who had undergone this operation, I frequently observed the limb half flexed upon the thigh. I could not learn whether this position was the choice of the patient or surgeon, or whether it constituted an accident in spite of the operator. My patient died on the 18th day, a very abundant suppuration having established itself. In regard to operating upon the upper extremity of the femur, the diagnosis is somewhat difficult, for if the disease extended to the bones of the pelvis it is obvious there would be no use in it. It is a long time since I performed resection of the tibio-tarsal joint, which I have done three times in all.

The joints of the lower extremity, however, have a greater tendency to anchylosis than those of the upper. It is very rare for this to occur after excision of the shoulder or elbow-joints. This is a vast advantage, especially as regards the elbow, and it would almost seem that the foresight of nature accomplishes the desires of the surgeon. The extremities of the bones become rounded; their inequalities are effaced, a sort of newly-formed cartilaginous crust covers them; the muscles contract new adhesions; and lastly, the movements, although somewhat anormal, and less extensive than in health, are established.—*Gazette des Hôpitaux.*

### Discharge of Serous Fluid from the Ear in Fracture of the Base of the Cranium.

This fatal symptom has formed a subject of frequent discussion in the French Medical Societies of late, and several papers relating to it have been published in recent numbers of the *Archives Générales. M. Laugier, of l'Hôpital Beaujon,* claims being the first person who directed attention to it in 1835; since which time many cases have been recorded. In 1839, he wrote of it as follows :—

" It consists in the flowing from the ear of a greater or less quantity of an aqueous fluid, at first lightly tinged with blood, but soon after limpid and colourless, being in fact the serosity of blood effused into the cavity of the cranium, between the dura-mater and the bones. The motions of the brain gradually express it from the coagulum, which is thus reduced in a few days to a thin layer, no longer capable of exerting compression upon the brain. The flow of this fluid, considered by itself, independently of other phenomena, indicates—1. The existence of a fracture of the petrous portion of the temporal bone.—2. That this fracture is a mere fissure; for, when a wider separation of its borders occurs, it does not act as a filter, but allows the entire mass of the fluid blood to pass through.—3. The presence of effused blood upon the fissure of the petrous portion, an important indication, if symptoms were such as to render the use of the trephine requisite."

M. Laugier observes, that all subsequent experience has shewn the correctness of the two first conclusions; but that the source of the fluid has been disputed both in France and England. Thus it has been urged, that the *quantity* of fluid discharged is far too considerable to allow of the supposition that it proceeds from the compression of the effused blood. From 12 ozs. to 24 ozs. have been collected. M. L. does not pretend that all this is obtained from the serum of the effused blood; for, in all wounds of soft parts or bones, there is always much serum poured out until suppuration is established. We see this after amputation, the abundance of the serum discharged in these cases being considered by Dupuytren of importance, as showing no danger of secondary hæmorrhage existed. M. L. however, admits that the analyis hitherto made of this fluid shows that it differs much from the *composition* of the blood, in the absence of albumen and in the presence of too much chloride of sodium.

Various other explanations have been proposed. Thus, the discharge has been attributed to an augmentation of the naturally trivial quantity of fluid contained within the labyrinth—the *liquor cotugni.* The quantity discharged would seem sufficient to refute this opinion; but it may be also stated, that the fracture, in some instances, does not extend into the labyrinth, and in other cases it has done so without any larger secretion taking place. Guthrie states the fluid probably proceeds from the sac of the arachnoid; but, in none of the cases observed in France, have the arachnoid or dura-mater been ruptured. M. Bodinier has of late stated his belief that, owing to the effusion of blood between the dura-mater and bones, the arachnoid fluid penetrates its membrane by *exosmosis,* and endeavours to justify his opinion by some not very satisfactory experiments on the dead body. It is to be observed, also, that in one case in which the discharge from the ear was very abundant, there was no effusion of blood between the dura-mater and the bone. M. Laugier, therefore, reiterates his opinion, that a *fissure* always exists in the petrous portion of the temporal bone, extending from the cavity of the cranium to that of the tympanum, or to the external auditory canal, and that the fluid is furnished by the effused coagulum, and by the vessels torn through in the solution of continuity.

*M. Chassaignac,* in a communication to the same journal, offers another explanation. He observes that, of all the bones of the cranium, the petrous portion of the temporal is in most intimate connexion with the large venous trunks and sinuses. The parietes of these vessels, which are attached to the bone, are very thin and delicate, so that the slightest fissure even would open into them; and, owing to the numerous and irregular points of contact between the sinuses and the bone, it is difficult to believe any portion of the latter can be fractured without the former becoming implicated. The details of a case which fell under his care are very minutely given, and the following general conclusions drawn.—"1. To appreciate the nature of these cases, we must examine the condition of the sinuses. To do this, we must slit up the non-adherent walls of each; and, after washing, carefully examine the adherent parietes from within.—2. The theory which indicates the spino-cephalic liquid as the source of this aqueous discharge, is in contradiction with those cases in which the fracture does not penetrate the meatus auditorius internus; it supposes a constant laceration of the meninges, which is often absent; it is supported by very doubtful experiments, and it supposes the rupture of the arachnoid layer serving as a sheath to the auditory nerve, which has not yet been demonstrated.—3. The petrous portion surrounded on almost every side by copious supplies of blood, is that of all the cranial bones, around which the greater number of venous channels is concentrated. Moreover, the sinus of the jugular vein, which contains the largest collective portion of blood in the cranium, is in such a relation to the petrous bone, that a fracture or fissure of the bone may easily induce a small rupture of this vessel.—4. The periosteal walls of the sinuses being much thinner and tenser than the meningeal walls are more easily torn.—5. It is almost impossible to suppose a fracture can occur in any part of the petrous bone without implicating a sinus.—6. It is then a rupture of the adherent wall of the sinuses, which allows these cavities to furnish the aqueous fluid seen dropping from the ear—the colourless portion of the blood separating when the fracture is very narrow, the coloured portion passing through when the separation of the edges of the fracture is wider."

M. *Robert*, in a communication subsequent to the above, states, as the result of the examination of the anatomical conditions of the cases observed by himself, and related by others—"That an abundant aqueous discharge from the ear, observed in fracture of the skull, indicates the existence of fracture across the middle portion of the petrous bone, involving the internal meatus, the labyrinth, and the inner wall of the tympanum: and complicated by the rupture of the membrana tympani." He believes the duplicature of membrane passing in with the auditory nerve is ruptured, and in this way the cerebro-spinal fluid discharged. He found such rupture during the careful examination of a case; while chemical analysis proves that the fluid discharged from the ear, and the cerebro-spinal fluid, are identical in composition.

In another case, related at great length, a copious aqueous discharge from the *nostrils* was observed. After death, a fracture was found, implicating the sella turcica, rupturing the arachnoid of the pituitary gland, and opening into the sphenoid sinuses.

M. *Kuhn*, of Niedermann, having seen an account of M. Laugier's cases, transmitted to the *Gazette Medicale* the history of one he met with a few years since. A boy, aged four years, jumping from a window eight feet high, fell upon his head, producing a contusion a little above the right meatus auditorius, without, however, any solution of continuity of the scalp. Paralysis of the lower extremities followed: he vomited much, and blood flowed from the ear; but his intellectual faculties were unclouded. Six hours after the accident, the blood from the ear was replaced by an aqueous fluid, which continued to drop from the passage for four days, so that it was calculated that between *three and four pints* must have in this way distilled. On the fourth day, the issue of this fluid suddenly ceased, and the child complained of violent pain in the head. This was of short duration; the paralysis, also, became less complete, and the child, in a few days, became entirely well, and has continued so ever since, with the exception of slight deafness.

### On the Cure of Eruptions of the Head and Face in Children.

M. *Trousseau* makes some interesting remarks, in his *Journal de Medicine,* upon the rules that should guide the practitioner in endeavouring to heal the eruptions, sores, &c. which affect the head and face of young children. To avoid circumlocution, we will employ, in the extracts we make from the paper, the term by which these are designated in France—*les gourmes*—equivalent to our appellation "breakings-out."

It is a popular opinion that danger attends the attempt to heal these, and this is sometime true when their manifestation is connected with a morbid diathesis. Others, however, unconnected with this, do much mischief, and should be healed at once. A diathesis may be acquired or congenital; and the *suppurative diathesis* is that which of all others is most evidently acquired. The "*gourmes*" are, indeed, generally one of the manifestations of this; while in other cases the *dartrous* diathesis, which is usually hereditary, plays an important part in generating the eruption. The form of the "*gourmes*" will vary according as one or other of these prevail. Impetigo, ecthyma, impetiginous eczema, intertrigo, furunculus, superficial phlegmon, and ophthalmia, are more especially connected with the suppurative diathesis; while, lichen, psoriasis, eczema rubrum, pityriasis, favus, and chronic inflammation of the eyelid, are more often dependant upon the dartrous diathesis.

1. When, from distress, neglect, or other cause, a superficial phlegmasia becomes in the course of several months converted into a suppurating sore, in the groin, behind the ears, or upon the scalp of the child, the economy, which at first suffered from the presence of an useless discharge, accustoms itself to it to such an extent that, although its suppression at an early period would have been very advantageous, this must now be accomplished cautiously, or disease and ill-health will result. 2. Again, when an impetigo suddenly develops itself in a child previously in ill-health, and becomes chronic; the health may become manifestly improved as long as the eruption continues. It is evident that, for a certain period at least, it should not be meddled with, and even then that its cure should be very cautiously undertaken. 3. The development of "*gourmes*," may be the signal of serious disorders in a child prior to this in good health. In this case their cure, if fever be present, should be set about at once without any fear of the pretended effects of a retrocession. 4. When a child's health is good, we must endeavour by every means to prevent the establishment of the "*gourmes*;" for, if suppuration be accidentally established, it may give rise to other suppurations—in fact, generate a suppurative diathesis. This diathesis again may manifest itself, not only on the skin and mucous membranes, but also in the internal organs; and thus, in children suffering from "*gourmes*," variola, rubeola, scarlatina, &c. are always more fatal. 5. When the "*gourmes*" invade important parts, as the eyes, nasal fossæ, auditory canal, &c., we must use every means to prevent their extension.

*Treatment.*—The superficial *excoriations* which are found behind the ears and between the folds of the skin in gross children, usually arise from negligence, and often disappear upon the mere observance of cleanliness. Soapy baths, dusting them with lycopodium, or the interposition of lint moistened in olive oil, usually suffice to dry them up: but when they are obstinate, white precipitate ointment (ʒj ad ʒx axung), or Galen's cerate, may be employed. Frequently, to cure the intertrigo behind the ears, it suffices to take care that the string of the cap be not too tightly tied, or to prevent the surfaces of the skin from coming in contact with each other.

*Impetigo, impetiginous eczema,* and *ecthyma,* in their *acute* form, require special treatment. Dr. Trousseau, regarding the two first as true eruptive fevers, just as scarlatina, variola, &c., is careful in not suppressing them too rapidly, although he does not encourage their development. So far from this, believing with Sydenham, that our object should be to prevent eruptive diseases becoming confluent, he prescribes prolonged baths, abstinence, acid drinks, and mild laxatives. The children are not to be too much covered up nor to be kept in bed. Excessive cleanliness is to be observed, and great care taken that they do not scratch the pustules and diffuse the disease with their nails over other portions of the body. When the febrile action has ceased, we have to do with a mere local disease, and must get rid of it as soon as possible. Unfortunately, however, impetigo oftentimes succeeds to measles and scarlatina; in which case, our proceedings must be more circumspect. If the impetigo be too rapidly healed, in this case, the lungs or some other internal organ will very probably become diseased, we having thus destroyed the revulsive affection of the skin which acted as a preventive, or as a curative if they were already affected. There are circumstances, however, in which such caution would be misplaced. Thus a violent inflammation of the ocular mucous membrane may spread to the eye itself, or a very severe eczema behind the ear may give rise to dangerous or even fatal enlargement of the cervical glands. In both these cases we must at once cure the eruption, as it gives rise to greater evils than we have reason to fear from its repercussion.

When the *impetigo* and *eczema* become chronic, and the condition of no internal organ causes alarm, I treat them with baths, ointments, lotions, purgatives, blisters, or depuratives. *Alkaline baths* are the best of remedies when the disease is attended with itching. To 75 or 100 quarts of water I usually add from 12 to 20 drams of subcarbonate of soda or potass. These baths most effectually clean the skin, soften the crusts, and relieve the pruritus. The dreadful suffering this last causes proves its relief alone is no slight advantage. With a solution rather stronger than that employed for the baths, lotions may be made and locally applied two or three times daily. These baths are suitable for the *dry forms* of *eczema*, for *lichen*, and for *pityriasis*. But when the *eczema* is very *acute*, and is accompanied by great redness and abundant discharge, *mercurial baths* are to be preferred. I prepare these by adding to 50 or 70 quarts of water three or four scruples of corrosive sublimate, dissolved in 1 oz. or 1½ oz. of alcohol. I have used these baths for fourteen years in every variety of dartrous affection of the skin with the greatest advantage. Some practitioners consider them dangerous, but I order about a thousand annually, and even for women in the weakest state and children of the earliest age, without ever seeing any accidents result from their employment. I have had children placed in these baths, half the skin of whose body had been destroyed by eczema, and no injurious absorption of the mercury has taken place, while the epidermis has become re-generated in a few days. Very young infants should not be kept in the bath more than a quarter of an hour at the farthest, but those who are more than a year old may be retained in for half an hour. The severest forms of *eczema, lichen, erythema*, and *impetiginous eczema* soon yield to these baths, and they form the most appropriate treatment for the *syphilides* of infancy. In simple, chronic *impetigo*, I find *sulphureous* baths, formed of 1 or 2 drams of sulphuret of potash to 50 or 70 quarts of water, best. But they are especially indicated in children covered with furunculi or little sub-cutaneous abscesses. The action of these baths is no doubt chiefly topical, for ointments composed of the same materials and applied to circumscribed spots are as useful; but when we find the alkaline baths correcting acid urine and the mercurial baths relieving syphilis, it is evident that some portion of their material is absorbed, as is also shewn by the odour which the sulphureous baths impart to the secretions. Indeed, experience has proved the efficacy of alkalis and mercurials, taken internally, in moderating the dartrous diathesis, which manifests itself in herpetic eruption.

When the affections of the skin are very limited, *lotions*, composed of the same materials, in larger proportions than in the baths, may be substituted. The strength of these must depend upon the susceptibility of the skin, and condition of the lesion; but the practitioner must not be afraid of using them pretty strong, as the temporary irritation they excite is often advantageous to the affection. In the treatment of "*gourmes*" of the hairy scalp, the sulphuret of potassium may be employed in such strong solutions as to be almost caustic. The *temperature* of these lotions should be as *high* as can possibly be borne. This may seem strange advice at first, but doubtless much of the efficacy of the vapour-bath in cutaneous affections depends upon the great heat thus produced, and the success attendant upon the employment of infusions of simple herbs by empirics in like manner results from their using these very hot.

Among the *ointments* those containing *mercury* occupy the very first place. White precipitate and calomel are usually to be preferred to red precipitate; but nothing absolute can be stated, for in apparently identical affections, sometimes the one and sometimes the other preparation proves most efficacious. The two former may be used in the proportion of one part to five or ten of cerate, and the red precipitate half as strong. In some children lard, and in others cerate, forms the best vehicle. In some diseases of the hairy scalp, alkaline or sulphureous ointments are preferable to the mercurial ones, and this is the case especially in the moist and scabby forms. In the dry and squamous forms, ointments formed of mercury, of pitch, or of sulphate of copper, are highly useful. But I cannot too often repeat, that we must try various means, and neither allow ourselves to be too much encouraged by former success, or discouraged if we find a remedy useful in some cases of no avail in others. Even for the same disease, the practitioner should always be provided with a certain variety of remedies, which will all, some day or other, be required.

I now come to the consideration of the employment of *blisters*. And first, let it be observed that a substance, such as Burgundy pitch, croton oil, or mercurial ointment, which when applied sometimes gives rise to the production of a local crop of vesicles, occasionally also leads to a *general* eczema, first acute and then chronic. This is a rare occurrence in men, rather more common in women, and very frequent in children. A few months seldom pass without my seeing, in hospital or private practice, an acute, simple or impetiginous eczema attack children, after the unavoidable employment of a temporary blister in pneumonia. Generally the disease assumes a chronic character; and if we consider that, up to this time, the child was not the subject of any cutaneous affection, we must admit the blister has been at least the occasional cause of its production. Seeing, then, that in a healthy skin a blister may develop a chronic cutaneous affection, ought we attach much importance to this means for the treatment of "*gourmes*," and rather ought we not reject it in the majority of cases? I have now in my wards a young child who, when the subject of a slight lichen upon some few points of the skin, was ordered a blister by its attendant. A few days after, the arm to which this had been applied was covered with eczema, which quickly spread over the rest of the body. I have frequently, in obedience to routine or theory, applied blisters to children affected with "*gourmes*," but have often repented doing so, and seldom seen benefit result. Believing, then, blisters only cause additional irritation without relieving that already existing, I proscribe them in cutaneous affections; but I employ them in treating the "*gourmes*" of the mucous membranes. Experience has often shown me disease behind the ear, or of the hairy scalp, alternating with ophthalmia or chronic eczema of the nasal fossæ, as if the two affections were incompatible. In this case, a blister to the arm is generally useful, although sometimes the derivation will not establish itself in the direction chosen by the attendant, but obstinately tends towards its original route. We may leave the blister on the arm, at the same time endeavouring to encourage the fluxion where it seems most willingly and beneficially inclined to place itself. But if blisters are of use in the cure of these, so to say,

*alternating* "*gourmes*," they are not so in "*gourmes*" resulting from *propagation*. Thus, we may often see an impetiginous eczema gradually invade the forehead, eyelids, conjunctiva, the rest of the face, and penetrate into the nose. I call this *propagation*, and in such a case blisters are of no avail. But if an ophthalmia replaces the eczema of the skin, which in its turn acquires predominance when the ophthalmia is relieved, I call it *alternating* or *compensating*, and here blisters are in general useful. If they are useful here, they are imperiously demanded when a bronchitis, an enteritis, a pulmonary, or intestinal catarrh is set up, and alternates with the cutaneous "*gourmes*;" for all these are but other manifestations of the same diathesis which a true pathologist must never overlook.

To decide upon the exhibition of *purgatives* is also somewhat difficult. The popular idea is, that these medicines constitute our sheet-anchor in treating "*gourmes*." If a somewhat severe diarrhœa occurs in a child subject to these affections, we observe on the very first day the eruption becomes paler, and if it continue, the inflammatory fluxion entirely disappears, and the cure may be effected without any topical remedy. If, however, the diarrhœa is naturally, or under the influence of medicine, arrested, you find the cutaneous affection almost immediately take on all the marks of activity it had lost. So that the antagonism between the skin and the gastro-intestinal mucous membrane is evident enough. With some practitioners, an artificial and spontaneous diarrhœa are the same things—in both, there is an intestinal flux. But the observer sees things differently. In spontaneous diarrhœa all the economy is prepared for this new fluxionary movement, and when it is established, it draws within its sphere of action a multitude of secondary vital acts. In artificial diarrhœa the economy resists the cause provoking it. There is doubtless a flux from the intestinal canal established; but it is isolated, all other acts of the economy retaining their independence. Compare the condition of the man who becomes the subject of a diarrhœa with his who takes a bottle of Seidlitz water, observe the exhaustion and *malaise* of the one, and the little inconvenience which a much greater number of stools causes to the other. A woman has not her menstrual discharge, or a man his hæmorrhoidal flux at their usual period, will the taking away a far larger quantity of blood than that usually lost from the vulva of the one, or the anus of the other, have the same effect on the economy? Some persons are affected several times in a year with an erysipelatous swelling of the nose or ear, substitute for such spontaneous irritation that produced by a large blister, and see if the effect will be the same. In a spontaneous act there is such a condition of the economy, that every function is in some measure subordinate to the actions about to take place, which can hardly ever be the case when the effect is sought to be produced by a therapeutical agent, unless indeed the indication has been well prepared and skilfully seized.

I have said enough to show that we must not judge of the influence which a purgative will exert by that which a spontaneous diarrhœa produces. But, if in lieu of the transitory action of a purgative given from time to time, we produce effect from day to day, or almost continuously; or again, if a temporary action be very energetic, and frequently renewed, we may produce results less marked, it is true, than those proceeding from spontaneous diarrhœa, but yet considerable enough to be of great importance to the practitioner. It remains to enquire whether a plan so acted upon is applicable to ordinary cases. I reply, it is not. It is dangerous for young infants, whether they are at the breast, or have been weaned. Gastro-intestinal phlegmasiæ, at this age, are of a grave character, whether considered as preventive of the active nutrition so requisite at this period of life, the acute, and often fatal affections they give rise to, or the chronic ailments they predispose to. Purgatives, to be of service in "*gourmes*," must be active, and it is easy to give rise to greater disorders than those we are seeking to combat. Such precautions are not required for adults, adolescents, or even for children above their third year, in whom these gastro-intestinal phlegmasiæ are established with difficulty, usually exempt from danger, and easily curable. If in an infant a slight diarrhœa, which had caused neither exhaustion or wasting, and yet had much improved the condition of the "*gourmes*," becomes arrested, we must endeavour, by the aid of purgatives, to reproduce it, and maintain it as nearly as possible in the same state it had previously existed in.

Various vegetable ptisans have acquired a reputation as *depuratives*, and many of these, as bitter-sweet or wild pansy, and also chicory-juice, are very useful adjuvants when taken for a long time by the children who have passed their first infancy. But I must protest against the employment of *cod's-liver-oil* and *hydriodate of potass* to this end, even when the "*gourmes*" can be traced to a scrophulous origin. I have almost always found these two therapeutical agents produce vesicular and papular eruptions; and, during the treatment of rickets, I have frequently been obliged to suspend the administration of cod's-liver-oil, because the skin has become covered with eruptions sufficient in many cases to excite considerable febrile action.

---

### On the Use of Galvanism in Lumbago, Sprains, and some other Painful Affections of the Muscles and Joints.—By M. Raciborski.

M. Raciborski observes that the utility of Galvanism in paralysis of particular nerves is well known, and that Magendie has proved by many recent cases its service in neuralgia generally, but especially in that of the branches of the fifth pair. Having witnessed many successful applications of this kind, mostly in the wards of M. Bouillaud, the author was led to believe the employment of galvanism might be advantageously extended to other affections characterized by violent pain and the absence of signs of inflammation, as muscular rheumatism and *lumbago*. His experiments have been highly successful, the suffering of this last painful affection being frequently forthwith relieved, after the patient had long tried other remedies in vain. The same may be observed of *rheumatism* affecting the muscles of the extremities. It is not easy perhaps to state the *modus operandi* of the remedy; but it would seem to be by directly subduing the *pain*, which prevents the contraction of the muscles, that galvanism produces the instantaneous relief seen in some cases. "Certain it is that, in many cases, we have applied galvanism with some success, even to painful swellings of the knees, rendering walking, if not impossible, at least very painful. Certainly galvanism did not cause the swelling to disappear, but the pain became dissipated, or so diminished, as to allow the patient to walk about. We do not doubt that the forced contraction which the galvanic shock produces in the fibres of the muscles, rendered motionless by the rheumatism, must contribute considerably to the good effects derivable from this means."

Four or five cases are given which were relieved almost immediately by galvanism, or rather, perhaps, we should call it galvanic acupuncture, inasmuch as needles were inserted in the parts where pain prevailed, and then brought in contact with the galvanic battery. A very few shocks, which usually themselves caused considerable temporary pain, sufficed to give relief, and enable the patient to exert muscular action without suffering. One or two of the cases seem to us, however, to have had all the characteristics of hysteria—but this matters little, inasmuch as an effectual means of relieving the pain of that troublesome affection is a *desideratum*.

"Since our notes were taken, we have had other opportunities of applying galvanism in analogous cases, and always with the same success; but, as at present we merely desire to draw the attention of practitioners to this new mode of treatment, we need not extend the paper by citing their particulars. Nevertheless, we cannot terminate it without signalizing the admirable effects which galvanism produces in the treatment of *Sprains.* Every one knows that a sprain, although apparently a slight affection, often exacts much time for its cure. When it implicates the ancle or knee, it is not uncommon to see patients deprived of the use of their limbs during two or three months. It is the violent pain felt upon the slightest motion of the part (we are speaking only of simple, uncomplicated sprain), which retards the cure. The other symptoms are of little consequence, and are usually dissipated promptly. Now, just as we have seen in lumbago, so in sprain, galvanism relieves this pain instantly, and allows the patient to walk without lameness."

M. Raciborski suggests that the galvanism may act by restoring the contraction and tension of the fibres of the articular capsule (and perhaps those of the tendons) which had been inordinately distended and elongated by the accident. —*Gazette Medico-Chirurgicale.*

---

### Iodine Injections in Chronic Articular Dropsy (Hydarthrosis.)

M. Jules Roux (of Toulon), having sent to the Academy an account of the successful treatment of a very large scapulo-humeral hydarthrosis by iodine injections, *M. Velpeau,* to whom the lot of reporting upon it fell, entered into the consideration of the management of *hydarthroses* generally, and his observations gave rise to a lively and prolonged discussion. We may first give a brief summary of M. Velpeau's remarks. He observes that many practitioners, having experienced the uselessness of topical remedies, such as lotions, ointments, &c. in these cases, have endeavoured by *general treatment* to effect the removal of the fluid. *Tartar-emetic and Calomel* have been the substances usually resorted to. M. Gimelle having observed the good effects of *Tartar-emetic,* when given in pleuritic, peritoneal, and other effusions, tried it in these, and with great success. M. Velpeau, however, in his trials, has found it of little, or at all events, only of temporary avail, although given in such large and long-continued doses as to risk exciting serious inflammatory action in the mucous-membranes of some subjects. *Calomel,* again, in numerous trials by exciting purgation or salivation, has induced a large absorption of fluid in a short period, but when its action has ceased, the effusion has been reproduced as abundantly as ever. *Very large* (*monstres*) *Blisters,* have also frequently been essayed by M. Velpeau. He has, during the last 15 years, employed these for a variety of acute and chronic inflammatory affections, not with a view to obtain a derivative action, but a positively depletory one, by inducing a great discharge of serum, which *large* blisters alone can produce. The general results he has obtained, he intends communicating shortly, and confines his attention now to the disease before us; and in this, he says, scarcely a week passes without exhibiting their advantage. He clothes the whole joint, however large, in a blister, which extends beyond the margin of the disease, repeating it, and in some cases leaving the part alone during the intervals, and in others dressing it with iodine or mercurial ointment, or applying methodical compression. It is often not until the blister has dried up that the benefit is perceived; the first blister sometimes, indeed, seemed only to increase the mischief. The suffering produced by large blisters is not much greater than that caused by small ones, nor do these necessarily irritate the urinary passages, or induce fever. They constitute then a most valuable means of treating hydarthrosis, whether in its acute or chronic stage. They are, also, of use when the disease is only symptomatic, and may, if desired, be employed consentaneously with general measures. They will not, however, cure every articular dropsy; that of some joints, as the shoulder and the hip, seeming to resist treatment more than others. Besides, some patients are unable to bear their application. Other means have to be resorted to in such cases, and among these are

*Puncture.*—In the case before us, M. J. Roux did not hesitate to let out a large quantity of fluid from the shoulder-joint by puncture, and many examples show us that this is by no means the dangerous operation it is stated to be by high surgical authorities. It should be performed with a small trocar, armed with a *cylindrical* canula, when the wound cicatrizes at once, and no inflammation follows. Unfortunately it is rarely successful, the diseased surfaces of the joint remaining unmodified. Finding the fluid re-accumulate, M. Roux again removed it, and threw *iodine injection* into the joint. This application of the treatment of hydrocele to this disease of the joints is no new suggestion; but the accidents it has given rise to when practised formerly, led to its being disapproved by Boyer, and the other high authorities. But in Boyer's cases the joint was not opened by puncture, but incision, and the injection not thrown in at once, as in hydrocele, and the puncture allowed to cicatrize, but thrown in from day to day, just as if you would cleanse out the cavity of an abscess. M. Velpeau has long been in the habit of treating the varieties of hydrocele in communication with the peritoneum with the same success as the ordinary disease. The inflammation has not been propagated beyond the parts with which the iodine injection has come into contact; but then he has always taken care to throw this in through a small puncture only; and in this way, he states, opening into joints produces little inflammation. He attributes very little importance to the *admission of air*, so much dwelt upon by the advocates of sub-cutaneous incision; and has frequently observed it within the tunica vaginalis after the operation, without any evil resulting. In regard to the fears of *anchylosis* being induced, M. Velpeau believes, from his researches upon the structure of serous membranes, that these are capable of reproduction after the junction of opposing surfaces.

"The most powerful argument for using iodine injections, is the impunity with which I have been enabled to throw them into almost every serous cavity of the body. During the 12 years I have used this fluid, I have employed it upon near 300 patients affected with hydrocele; in cysts in all parts of the body, the interior of the vulva, and the female pelvis, in hernial sacs; in those seated in the groin, the iliac fossa, and the breast; in *goitres;* in different species of serous and sero-sanguineous cysts in the hyodean, parotidean, and sterno-mastoidean regions; in the bursæ mucosæ of the dorsum of the foot, the malleoli, and the patella; iu the synovial cavities of the tendons of the foot, the ham, the wrist, and the elbow, &c., and all this with such fortunate results, that it is impossible for me henceforth to fear the consequences. The knee was the first joint in which M. Bonnet and myself employed iodine injections for hydarthroses."

Many members of the Academy took part in the discussion. M. *Roux* (of Paris) considered M. Velpeau's assertion, that inflammation never followed simple puncture for *hydrocele,* as contradicted by all experience, and that his substitution of solution of iodine for vinous injections in treating it, to be any thing but an improvement. M. R. has employed wine in from 12 to 1500 cases, and has only met with five or six accidents, and that twenty years ago. He has yet to learn that iodine injections are harmless, and that they are less efficacious, is seen in the number of relapses which occur, these yielding easily enough afterwards to vinous injection. Moreover, he could not agree with M. V. that an injection may be efficacious without inducing adhesion of the opposite surfaces. As to applying this method to *hydarthroses,* it is not advisable, seeing they may be cured by more safe means.

M. *Blandin,* also, opposed M. Velpeau's conclusions. He stated that a simple puncture of a joint containing fluid by means of a trocar, is by no means dangerous, but that the *admission of air* may produce the worst consequences: M. V. has not alluded to two means of treating hydarthrosis which usually prevent the necessity of having recourse to any other. The one is inducing salivation by the local application of *mercurial ointment* to the part, and the other, the envelopment of the joint by the *starch bandage*, either or both of which methods M. B. has found very successful. The substitution of iodine injections for vinous, in *hydrocele*, is unadvisable, inasmuch as the latter are, when properly thrown in, quite innocent and efficacious, whereas the former frequently fail. In employing these in *hydarthrosis*, M. V. says he has met with no serious accidents, but certain it is he will do so ere long, if he persevere in such a course.

M. *Gerdy* stated that M. Lugol and others have long employed injections for dropsical joints, but that he has never had recourse to any such proceeding himself, believing it to be a dangerous one. He has known very bad consequences result; and one of M. Velpeau's own patients died poisoned by the iodine absorbed. So, too, in *hydrocele*, a man died at Calcutta after iodine injections, and as the same inconveniences which occasionally occur after vinous injections occur after iodine ones, while their efficacy is not so great, why should their substitution be attempted.

M. *Jobert* could not admit that a greater number of relapses follow the iodine than the vinous injection for hydrocele. He has met with but one out of 75 cases in which he has used it. Comparing the one with the other the vinous gives rise to much more pain and a higher degree of inflammation, sometimes terminating in abscess—so that the substitution of the iodine is a gain to surgery. He has also employed this with advantage in other cases, as chronic abscess, hydatid tumours and cysts, and tubercular cysts. In a patient who had a vast abscess of the neck, after twice evacuating collections of matter, he threw in 8 ozs. of the iodine injection with the effect of producing adhesion of the walls of the abscess.

M. *Berard* observed that, although at first prepossessed against the iodine injections in *hydrocele*, upon trial he became so convinced of their superiority, that he has since employed no other. He has also used them in encysted dropsies of the cord, in goitres, in hydatid cysts of the wrist, and in articular dropsy. He employs M. Velpeau's proportions of equal parts of water and tincture. He has never met with any general or local ill-consequence, the pain being slight in some cases, severe in others. In between 250 and 300 operations for hydrocele, he has met with but three relapses, which were relieved by a second injection. Success attended injections also in two cases of encysted hydrocele, and in two of encysted hydrocele of the neck: but in the two cases of articular dropsy they were inefficacious, amputation being afterwards required.

M. *Laugier* stated that he had had occasion to employ iodine injections for *hydrocele* a great number of times, and could only congratulate himself on the results. He had met with neither accidents or relapses, and considers the procedure more simple and precise than that of the employment of wine, which varies so in strength and quality.

M. *Velpeau*, in reply, denied that one of his patients had died poisoned by iodine. The operation was quite successful, when a sudden hæmoptysis carried him off, between two and three months after its performance. In reference to the *relapses* after iodine, we must not forget that these not unfrequently occur after vinous injection also. M. V. prefers iodine, just as he once did wine, because, after a trial of every substance which has been recommended, he finds it the best. The want of success of *Fricke* of Hamburgh has been cited: but this practitioner used an exceedingly weak solution, and re-operated in 10 or 12 days—a period far too short to judge whether success had occurred or not. Iodine does not require, like wine, that the *tunica vaginalis* shall be distended, and, in a great majority of cases, causes infinitely less pain, partly, no doubt, on this account. Encouraged by his first successes, M. V. has been enabled to apply this remedy to more and more serious diseases; and it is important to observe that, when it becomes accidentally thrown into the cellular tissue, it does not cause gangrene, as wine does; and various experiments have convinced M. Velpeau that it possesses the property of exciting a special inflammation, which does not terminate in suppuration.—*Bulletin de l'Academie.*

---

### On the Chlorosis of Adults. By M. Blaud.

"There is a disease, pretty commonly met with, which seems to me to have escaped the observation of most practitioners. This affection, of great danger if misunderstood, is the *Chlorosis of Adults.* I do not allude to that decoloration of the skin dependent upon some prior disease, such as hæmorrhage, intermittent fever, or some organic lesion, acute or chronic, &c.; but to an *essential idiopathic chlorosis,* proceeding from no other cause but such as produces it directly, that is a particular defect of hæmatosis, and which can only be relieved by re-establishing the normal condition of this function.

"Chlorosis is by no means peculiar to young girls arrived at puberty, and it is from want of attention to this fact, that many practitioners have mistaken it, when occurring in adults, for different organic lesions, which in fact had no existence. Some, seeing the pale or yellow skin, have declared it to be symptomatic of hepatic lesion, and others have attributed it to affection of the spleen. Some attribute the dyspnœa and palpitations to lesions of the heart or large vessels, and others see in these the symptoms of an asthma. Such different views lead to various plans of treatment, none of which are efficacious, the chlorosis, indeed, seeming to derive a new intensity from their operation. Chlorosis may attack adults of either sex, any age, and of any condition in life. We have seen subjects of it persons leading the most active and laborious lives, and others passing their time in luxurious idleness. The sober and the intemperate; those whom misery oppresses, and those who enjoy every abundance in life, may alike become its victims. So that the *predisposing* causes of the affection are as yet very obscure. The colourless and serous condition of the blood, the general debility, and languor of every function, seem to show that defective hæmatosis is the *proximate* cause. The *symptoms* are the same as in the chlorosis of young girls—such as the yellowish-green skin (the conjunctivæ preserving their normal whiteness), dyspnœa upon locomotion, *bruit de souffle* in the course of the carotids, &c. Still there are modifications noticeable. The colour of the skin is rather greyish or earthy, than yellow, in consequence of its rough or wrinkled state, especially in the face. The palpitations are more severe; while there is moreover a deep, insupportable *malaise,* sometimes accompanied by a disposition to suicide, and a more or less abundant anal hæmorrhage coming on at irregular intervals. With the general languor and disorder of the digestive organs, there is œdema of the extremities; and, if the disease be left to itself, a serous effusion into the cavity of the abdomen results from the atony of the absorbents. However severe these symptoms may appear, they never resist appropriate *treatment,* when this is not resorted to too late."

Of the cases he has met with, M. Blaud selects eight for description, four of them being women from 26 to 37 years of age, and the others, men engaged in active or laborious occupations. In most of these, various kinds of treatment had been employed before they came under the author's care, in the idea that organic disease was present: but he, guided by the general appearance of the patient and the absence of marked signs of such lesions, treated them all for chlorosis with complete success. He employed his own anti-chlorotic pills,*

---

* M. Blaud's pills are thus composed and administered. Let equal parts of which have attained so high a reputation in France, in every case; and complete cures resulted in periods varying from 10 to 30 days of taking them. He adds in a note, that long experience has convinced him that these pills are also a specific in *intermittent fever accompanied with œdema of the lower extremities,* which has resisted quinine and all other febrifuges.

---

### M. Reveille-Parise on the Employment of Cold Cataplasms.

Every one acknowledges that, in medicine and surgery, as in everything else, we frequently follow practices from habit, prejudice, or routine, in contradiction to the most evident and best-established principles. An example is found in the constant use of warm, so called *emollient,* cataplasms, in numbers of cases in which a high temperature is entirely opposed to the indications, which teach us to diminish the excitement and the activity of the circulation, by preventing the excessive development of caloric. Upon the occurrence of a wound or bruise we have recourse to refrigerants, in order to diminish as much as possible inflammatory reaction; but suppose this has already commenced, we then apply warm poultices. Caloric is the most powerful exciter of vital action, and to apply it in these cases is to follow in the steps of the homœopathists, without however adopting their infinitesimal doses. So far from being *emollient,* the great heat at which they are applied renders them stimulant. They may in a few cases act advantageously by augmenting the inflammatory action, and thus hasten on the formation of pus; but, in the great majority of cases, this is not the object which we have in view, but the removal or the diminution of inflammatory action.

M. R. P. cites some cases in which he substituted cold for hot cataplasms with the greatest advantage: among others, some instances of *panaris,* in which, after incising and disgorging the parts with tepid water, the greatest ease attended the use of cold cataplasms, while warm ones only aggravated the suffering. We employ cooling drinks and other means of lowering the temperature in fevers, and in eruptive diseases, and we know how hurtful heat is in an excited condition of the skin, as in pruritus; and yet we pursue an opposite practice in phlegmon, traumatic inflammations, acute rheumatism, &c. Formerly, warm poultices were applied in cases of ophthalmia, but judicious surgeons have long substituted cold applications; and Dr. Tanchou has also employed these with advantage in certain tumours of the breast, &c. threatening to degenerate into cancer.

The cataplasm, however, need not at first be absolutely cold, as it might excite pain in the morbidly sensitive inflamed surface, especially in women, children, and nervous or delicate persons. Many persons, from the effects of habit, can only gradually accustom themselves to bear it quite cold, the same application causing pain at first which afterwards produces ease. So that we must gradually adapt the means to the patient's sensibility. Moreover, a cold poultice must not be left too long unchanged, as it produces then a very disagreeable sensation. When the inflammation is active the poultice soon becomes heated, and,

---

sulphate of iron and subcarbonate of potass be finely powdered separately, and then intimately mixed. Beat them into a mass with tragacanth mucilage. Twelve grains form two pills. Take two morning, mid-day and evening, on the first, second, and third days: four morning and evening on the fourth, fifth and sixth days; four morning, mid-day, and evening, on the seventh, eighth and ninth days; six morning and evening on the tenth, eleventh, and twelfth days: and then six morning, noon and evening, until cured.

although some patients feel no speedy inconvenience from this, others, owing to the evaporation being impeded by the thickness of the cataplasm, are uneasy if it is retained for long. If, however, the patient complains of no uneasiness, the poultice may be left on with advantage for hours.

After the cataplasm has been applied for awhile, the patient usually feels much easier, the pain, heat, irritation, tension, and pulsation abating markedly. On examining the part, we find the redness much diminished, and oftentimes a pale patch here and there announces the commencement of resolution. Cold is in fact the most powerful of sedatives, and its action upon our tissues is far more efficacious than is usually believed. It prevents, by contracting them, the engorgement of the smaller vessels, it condenses the fluids, moderates the circulation, benumbs the sensibility, and diminishes irritation. Moreover, in the above gradual mode of employing it, we do not find that re-action of heat and redness which generally follows its sudden application to the surface. If, notwithstanding our care, suppuration does take place, this is always less abundant than when the inflammatory action is stimulated by hot poultices. The beneficial action of these cataplasms may also sometimes be increased by mixing medicinal bodies with them, such as a solution of acetate of lead, the various narcotics, &c. "In cases of inflammatory swelling and acute neuralgia, I have anointed the parts with belladonna ointment, and, leaving a layer on the surface, have applied over it cold cataplasms. This practice is almost certain to be successful, if the cause of the affection does not continue in operation."—*Bulletin de Therapeutique.*

(There can be no doubt that hot cataplasms are too indiscriminately employed, and indeed the practitioner usually contents himself with ordering a poultice without giving any directions as to its temperature. Patients look on with the same incredulity as did the host of the Satyr, when they are told the same means may prevent or hasten suppuration, according as it is applied in a cold or hot condition. We are surprised M. Reveille-Parise takes no notice of water-dressing, which is in most cases so elegant and useful a substitute for cataplasm.—*Rev.*)

---

### Aneurism of Bones. By M. Nelaton.

Several authors have noticed this disease, but many of the cases cited are evidently but examples of malignant tumours or serous cysts deriving pulsations, without direct communication with the arteries. True, simple, aneurism of the bones is in fact a very rare disease. It is especially found within the cancellous structure of the extremities of the long bones, the upper extremity of the tibia being beyond all other parts most frequently its seat. Aneurisms are however sometimes found in all the other long bones, as also in the flat ones, as in M. Roux's case of aneurism in the diploe of the parietal bone. In proportion as the tumour increases, the structure of the bone becomes removed, with the exception of a thin shell, and even complete perforation would sometimes ensue but for a thickening and fibro-cartilaginous transformation which the periosteum undergoes. In one of Scarpa's cases the articular extremity would have been completely separated from the body of the bone, but for the periosteum, which held the parts together. The trunks of the principal vessels undergo no alteration whatever; but the branches which enter the spongy structure of the bones are dilated, a vast number of them opening into the sac. The joint in the vicinity of the aneurism has always been found healthy. Breschet compares these to the erectile tumours of soft parts, but the analogy does not hold good; for, instead of erectile tissue, we have a true sac containing layers of coagula, just like ordinary aneurism; but supplied by many small vessels instead of one large trunk. Scarpa finding, in two cases he examined after death, a yellowish, semielastic substance under the periosteum, was disposed to believe that the early stage of the disease consisted of an erectile tumour, and that the aneurism, properly so called, was only formed at a later period, by a rupture of a vessel in this tissue. It is, however, possible that he mistook a pulsating cancerous tumour for aneurism. Cancerous degeneration is often seen in the vicinity of these aneurisms, and the affinity of the two affections may explain the frequent examples of relapse.

*Symptoms.*—Sometimes the pain and uneasiness of this disease is long in establishing itself; but for the most part it comes on quite suddenly, with a sense of cracking near the joint. After continuing two or three months a tumour is perceived. It is at first very small, and may escape notice; but after a while becomes prominent, the skin over it then becoming violet-coloured, and transparent, so as to exhibit the numerous subcutaneous veins. On examining the tumour we find it to be connected with the bone, and presenting different degrees of consistency at various points. Frequently, on pressing the more resisting portions, we are sensible of a sensation which has been compared to the crackling of parchment, or the breaking of an egg-shell, a sign dependent upon the depression and re-elevation of the thin osseous shell of the bone. One of the most characteristic symptoms is the well marked pulsations synchronous with those of the heart, and which are suspended when the principal vessel leading to the part is compressed. There is no *bruit de souffle.* The disease has always been observed in young persons or adults, and has, in different cases, been attributed to various acts of external violence; although, doubtless, the changes in the bone had already commenced. The *progress* of the disease is generally slow. Many of the patients have had these tumours for several years without their acquiring a very large size. Do they not, like other aneurisms, burst when they have reached a certain size? It is possible; but there is no authentic example of their doing so; for the ulcerations and hæmorrhages spoken of by some authors, probably arose from pulsating cancerous degenerations.

*Diagnosis.*—An aneurism of a bone may be confounded with one of the soft parts, the symptoms of the two being so very similar; and, before *post-mortem* examinations had explained the true nature of these cases, the mistake was inevitable. In the cases treated by Pearson, Scarpa, and Lallemand, the disease was supposed to be an aneurism of the articular arteries of the knee, or of the anterior tibial. The osseous aneurism forms one body, as it were, with the subjacent bone, a thin shell of which imparts a sense of crepitation; when the tumour is reduced by slow pressure, we perceive the loss of substance in the bone. The aneurisms unconnected with the bone are more mobile, and impart the *bruit de souffle* to the ear. A *malignant pulsating tumour* is distinguished with greater difficulty. The chief points are, that it cannot be partially reduced by pressure to the same extent as an aneurism, while it usually gives the *bruit de souffle* in auscultation.

*Treatment.*—It is a most serious disease, for if left alone it entirely destroys and renders useless the bony parts it is seated in; while an operation is dangerous, and frequently followed by a relapse. *Excision* of the diseased part is obviously impossible in most cases. M. Roux relates the only one on record in which it was performed by a surgeon at Geneva—a portion of the walls of the cranium being removed, the patient died in a few days. *Amputation* has been frequently performed, and perhaps is the only suitable operation when the disease has reached a certain point. The analogy existing between these and ordinary aneurism suggested to Dupuytren the employment of the *ligature.* He practised his operation in 1819, as did M. Lallemand in 1826. M. Roux, has recently published two other cases; and the author of this paper tied the crural artery about three months since. In Dupuytren's case a relapse occurred six years after the operation, rendering amputation necessary. M. Lallemand states that his case was cured, without however, furnishing any particulars of the condition in which the limb was left in. In one of M. Roux's cases, the brachial artery was tied for a pulsating tumour, at the extremity of the radius, but the disease, which was combined with cancerous degenerescence, was only temporarily arrested, and amputation afterwards was had recourse to. The second case, in which he tied the crural artery, was quite successful, and five months after, the tibia had almost recovered its normal condition. In M. Nelaton's own case, the pulsations at first ceased, but were soon reproduced, and the tumour is only a little less three months after the operation, than before this was undertaken. When the pulsation recurs, after being temporarily arrested by the ligature, we may, like Dupuytren did, have recourse to compressing the principal artery leading to the part, and even the sac itself.—*Gazette Medico-Chirurgicale.*

---

### M. Berard on Spontaneous Hæmatocele.

Boyer states that Hæmatocele is never spontaneous, but always results from contusion or operation, and the majority of cases that are met with in practice arise in this manner; but if the statements of patients are to be believed, cases occasionally occur in which blood is effused into the tunica vaginalis or cellular tissue of the of the scrotum without any known traumatic cause. The origin of the disease in such cases is as obscure as it is in many examples of effusions into the arachnoid, pleura, and other cavities. This origin of hæmatocele should be carefully borne in mind, as it may be confounded with much more serious diseases; for practitioners at present, on learning from the history of these cases that no external violence had been suffered, would be much more likely to refer the symptoms to encephaloid cancer than to their true cause.

Spontaneous, like traumatic hæmatocele, may be either *extra-vaginal* or *intra-vaginal.* The blood in either case may remain a very long period without being absorbed, a portion of it becoming coagulated into concentric layers, like that of an aneurismal sac, and the rest retaining its fluid condition. The effusion may be so considerable that the form, size, and consistence of the tumefaction much resembles degenerated structure. When the hæmorrhage is external to the tunica vaginalis the diagnosis is generally more easy, as it is less densely covered, and more easily assumes an irregular form not usually observed in affections of the testicle and tunica vaginalis. But when the effusion takes place within the sac, the testicle becomes entirely surrounded by the blood, and the epididymis is completely concealed by it, so that the actual condition of the testis cannot be ascertained. In such a case, if the skin is observed to be reddened and somewhat thin, the veins dilated, the pains of a lancinating character, the weight of the tumour considerable, and the sensation imparted to the fingers, resembling the semi-fluctuation of half-softened encephaloid, you may readily mistake the affection for cancer. Even an exploratory puncture may lead to error, if the needle only penetrates the coagulated blood.

Two cases are related. In the first (a lad of 15), of the *extra-vaginal* variety, the affection had only been ten days in acquiring the size of a turkey's-egg, and the testicle was found to be distinct at the upper part of the tumour. The diagnosis was therefore easy, and a free incision having evacuated the blood, a moderate degree of suppuration was established, and the patient was cured in a fortnight. The other (æt. 25), an example of the intra-vaginal variety, the swelling had occupied more than a year in reaching the size of a fist. The skin was distended, the weight of the tumour considerable, the veins on its surface somewhat dilated, and the pains were of a lancinating character. The swelling was not transparent and was heavier than a hydrocele. It was not fluctuating, but a softening could be felt here and there. Its surface was not irregular as that of scirrhus or mammelonated like tuberculous testis. The testicle could not be felt separately, a slight projection only being supposed to indicate the situation of the epididymis. An exploratory puncture was made at the lower part of the tumour, but no liquid flowed out. The existence of a sarcocele seemed to be well established, and an operation was proceeded with. But the error of diagnosis was discovered upon opening the tunica vaginalis, which was found to contain a large quantity of coagula, disposed in some situations in concentric layers, and about two ounces of fluid blood. The testicle was enveloped in the solidified effused blood, and was found to have retained its normal size and shape.—*Gazette Medico-Chirurgicale.*

---

### Professor Paul Dubois upon the Signs of Pregnancy.

We are glad to find by a recent Number of the *Gazette Medicale,* that this eminent practitioner is about to publish his work on Midwifery, which has been so long promised and so anxiously expected. Judging from the Chapter there printed it will prove of the useful and practical character his high reputation and large practice would have led us to anticipate. We have only space for a few of his remarks upon the condition of *menstruation* and the *breasts* in pregnancy. After quoting with approbation Gooch's useful observations upon the suppression of menstruation without pregnancy, he continues—

"That the menses also may continue although pregnancy exists is a fact now well established in science, and a year does not pass without my having occasion to point out to my clinical pupils well-marked examples of this exception. Although these anomalies have attracted the attention of practitioners for a long period, the exactness of the observations upon which they are founded has been denied by very high authorities, such as Denman and Hamilton. My father, also, whose experience was very extensive, and whose enlightened mind allowed him to respect opinions quite contradictory to those which he held, taught that the persistence of the menses was a sufficient reason to declare that the patient was not pregnant. Observation will not sustain these opinions; but when we find them expressed by men whose ability, experience, and judgment cannot be called into question, it is certain that menstruation during pregnancy must be at least a rare phenomenon, and that young practitioners singularly abuse their acquaintance with this exception, in admitting much too often, and almost always erroneously, the existence of pregnancy in women whose menses are not suspended. * * * * * * * * *

"If there are circumstances under which the mere consideration of the state of the menses would lead us into error, there are others in which it is of no avail. I have said elsewhere, that although the establishment, and to a certain extent, the regularity of this function, are favorable and almost necessary for fecundation, it yet must not be denied that this may occur in the absence of these conditions, of which I furnished several proofs. Women too have become pregnant without having been regular, or even being destined ever to be so. Others have become so after the natural cessation of the function; and others again, who have had long intervals between their periods, or in whom the menses have been suppressed by serious diseases. Lastly, others have become so during the temporary suspension incident upon suckling. These anomalies, however, do not, like the preceding, lead us to erroneously admit or reject the existence of pregnancy. They are not the cause of an erroneous opinion, but they may prevent our forming a correct one. Some women, again, by a freak of their organization are made aware of their pregnancy by the very sign which excludes the idea of it in others. Such are women who are not usually regular except when they are pregnant, or others, who, although regular, yet also meet with a discharge at an unexpected period whenever conception occurs. Deventer and Baudelocque have furnished examples of the first of these, and Desormeaux, Pusoz and others of the second."

*The Breasts.*—" We must remember that the swelling and sensibility of the breasts are very slight during pregnancy under certain circumstances, as in women of very delicate constitution or enfeebled health, in some of whom hardly any change occurs. More importance is usually attached to the modifications which the nipple and its areola undergo. These consist in—1. A tumefaction (which appears œdematous or emphysematous) of the skin of this part of the breast. 2. A development of papillary tubercles. 3. A more or less deep brown colour. The *humid and emphysematoid swelling* of the nipple and areola has seemed to me to be very well marked in some women, but inappreciable in most others, and on this account I do not think with Hamilton, that this is a very useful diagnostic mark. There is not the same difficulty with respect to the *papillary tubercles,* which are frequently developed in pregnancy, and easily recognized. Unfortunately, their value as a sign is diminished by the fact of my having observed them well developed in women who were not pregnant, most of whom certainly had had children, but several never had. Then in a very great number of pregnant women they are not to be perceived; and this fact has so forcibly struck me, that I have enquired of myself whether the difference in the observations made by Montgomery in Ireland, and mine in France, might not be attributable to a difference in the organization of the nipples in the subjects of our respective researches. This phenomenon taken alone is then far from having in my eyes the importance attached to it by the distinguished Dublin accoucheur. In its semeiological relation, *the brown colour of the nipple* seems to me to possess most interest, inasmuch as it is more easy of detection than the turgescence of the nipple, and its manifestation is much less exceptive than that of the tubercles. Still I must observe, this sign is sometimes wanting, especially in women with very white skin and fair hair; that as the colour is more or less persistent after the first pregnancy, it does not throw much light upon subsequent ones; and that, according to some good authors, it may be occasionally induced by other causes than pregnancy. As then neither of these conditions of the nipple taken alone, is a demonstrative sign of even a first pregnancy, does their co-existence possess more value? Montgomery believes that all these conditions of the nipple occurring in a woman who has never borne children present a positive indication of the existence of pregnancy, and his opinion seems to me quite admissible. I must add, however, that I do not regard their absence as an absolute negative sign; and that, even when they are visible enough to constitute a demonstrative sign, the pregnancy is usually sufficiently advanced to allow of its detection by more certain means than the examination of the areola."

---

### A Cheap Substitute for a Vapour-bath.

Dr. Serre (d'Alais) recommends the following means of inducing abundant transpiration. " Take a piece of lime about half the size of your fist, and wrap around it a wet cloth, sufficiently wrung to prevent water running from it. A dry cloth is to be several times wrapped around this. Place one of these packets on each side the patient when in bed. An abundant humid heat is soon developed by the combination of the lime with the water, which quickly induces copious transpiration—the effect of the apparatus lasting for two hours at least. When sweating is fully established, we may withdraw the lime, which is now reduced to a powder, and is easily removed. In this way, neither copious drinks or loading the bed with coverings is required."—*Gazette Medicale.*

